# Cyclic loading increases friction and changes cartilage surface integrity in lubricin-mutant mouse knees

**DOI:** 10.1002/art.33337

**Published:** 2012-02

**Authors:** Elizabeth I Drewniak, Gregory D Jay, Braden C Fleming, Ling Zhang, Matthew L Warman, Joseph J Crisco

**Affiliations:** 1 Alpert Medical School of Brown University and Rhode Island HospitalProvidence, Rhode Island; 2 Howard Hughes Medical Institute and Children's HospitalBoston, Massachusetts

## Abstract

**Objective:**

To investigate the effects of lubricin gene dosage and cyclic loading on whole joint coefficient of friction and articular cartilage surface integrity in mouse knee joints.

**Methods:**

Joints from mice with 2 (Prg4^+/+^), 1 (Prg4^+/−^), or no (Prg4^−/−^) functioning lubricin alleles were subjected to 26 hours of cyclic loading using a custom-built pendulum. Coefficient of friction values were measured at multiple time points. Contralateral control joints were left unloaded. Following testing, joints were examined for histologic evidence of damage and cell viability.

**Results:**

At baseline, the coefficient of friction values in Prg4^−/−^ mice were significantly higher than those in Prg4^+/+^ and Prg4^+/−^ mice (*P* < 0.001). Cyclic loading continuously increased the coefficient of friction in Prg4^−/−^ mouse joints. In contrast, Prg4^+/−^ and Prg4^+/+^ mouse joints had no coefficient of friction increases during the first 4 hours of loading. After 26 hours of loading, joints from all genotypes had increased coefficient of friction values compared to baseline and unloaded controls. Significantly greater increases occurred in Prg4^−/−^ and Prg4^+/−^ mouse joints compared to Prg4^+/+^ mouse joints. The coefficient of friction values were not significantly associated with histologic evidence of damage or loss of cell viability.

**Conclusion:**

Our findings indicate that mice lacking lubricin have increased baseline coefficient of friction values and are not protected against further increases caused by loading. Prg4^+/−^ mice are indistinguishable from Prg4^+/+^ mice at baseline, but have significantly greater coefficient of friction values following 26 hours of loading. Lubricin dosage affects joint properties during loading, and may have clinical implications in patients for whom injury or illness alters lubricin abundance.

Lubricin, a major component of synovial fluid and the articular cartilage surface, is a mucinous glycoprotein encoded by the gene *Prg4* (1). To understand the in vivo function of lubricin, lubricin-null mice (Prg4^−/−^) were generated. Prg4^−/−^ mice appear normal at birth, but develop precocious clinical, radiologic, and histologic signs of joint disease as they age (2). These features recapitulate findings in patients with the camptodactyly-arthropathy–coxa vara–pericarditis syndrome (CACP) due to hereditary lubricin deficiency. Based on in vivo studies in Prg4^−/−^ mice, lubricin was postulated to provide chondroprotection by acting as a boundary lubricant, a cell adhesion inhibitor, and a synoviocyte growth regulator (2, 3). While the precocious cartilage failure observed in patients with CACP and in Prg4^−/−^ mice indicates that lubricin is essential for chondroprotection, it is not known whether lubricin dosage that is below wild-type (Prg4^+/+^) levels, but not completely absent, can also impair chondroprotection. In vitro studies that measured boundary lubrication at different concentrations of lubricin, and clinical studies showing that lubricin levels fall in humans and other mammals following traumatic joint injury, suggest that a correlation may exist between lubricin concentration and chondroprotection (3–19). This work seeks to directly test this hypothesis.

Cyclic loading has been used in many studies to better understand the mechanical and material properties of articular cartilage (14, 19–22). Pin-on-disc systems rub cartilage samples against apposing cartilage plugs or man-made surfaces. Such systems have been used to collect information on friction, deformation, and wear, among other properties (19–21, 23, 24). In vitro pin-on-disc testing methods allow for focal tissue-specific investigation of articular cartilage; however, they are challenging to implement when studying whole-joint function and health. For this study, we developed an active pendulum system to apply cyclic loading to intact mouse knee joints in order to assess the role of lubricin in preventing cartilage wear.

We previously showed that mice completely lacking lubricin had higher coefficient of friction values and more extensive surface damage than wild-type mice (13). The objectives of the present study were to examine the effects of lubricin dosage and cyclic loading on whole-joint frictional properties and the surface integrity of articular cartilage in knees from wild-type mice (Prg4^+/+^), mice with one allele that expresses lubricin (Prg4^+/−^), and mice with no alleles that express lubricin (Prg4^−/−^).

## MATERIALS AND METHODS

### Specimens

In accordance with procedures approved by the institution's Animal Welfare Committee, hind limbs from 10-week-old (± 0.5 weeks) male Prg4^+/+^, Prg4^+/−^, and Prg4^−/−^ mice (n = 12 for each genotype) were disarticulated at the hip joint following euthanasia. The generation of these mice has been described previously, and they have been backcrossed onto a C57BL/6J background strain (1). Following disarticulation, skin, musculature, and connective tissue were dissected free, but joint capsules were left intact. Each paw was removed at the ankle joint, leaving just the femur, knee joint, tibia, and fibula. In order to mount specimens in the testing systems, the proximal femur and the distal tibia were rigidly potted with a urethane-potting compound (Smooth-On) in square brass tubes (6.25 mm wide and 25.4 mm long).

### Pendulum systems

**Figure 1:**
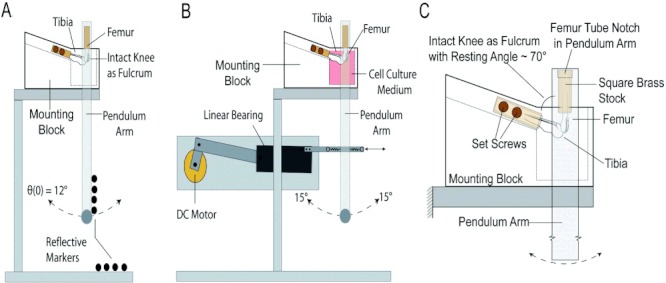
Schematic diagrams of the 2 pendulum systems used in the experiment. A, Passive pendulum system used to measure the coefficient of friction. B, Active pendulum system used for cyclic loading. C, Illustration of how each mouse knee joint was positioned in the mounting block. Color figure can be viewed in the online issue, which is available at http://onlinelibrary.wiley.com/journal/10.1002/(ISSN)1529-0131.

Two custom-made pendulum systems were used in this study. A passive pendulum system was used to determine joint coefficient of friction (Figure 1A), and an active system was used to apply cyclic loading to the knee joints (Figure 1B). Both systems included a platform with a specimen-mounting block (Figure 1C). In the passive pendulum system, the tube encasing the tibia was set in the mounting block and fixed with 2 set screws. The pendulum arm was fit to the proximal femur and weighed 50 gm, ∼2 times that of the mice used in this study. The femoral tube was attached to the pendulum arm by a square notch located in the top portion of the pendulum. The knee joint served as the fulcrum of the pendulum and had an equilibrium knee flexion angle of ∼70°, which is the mean flexion angle mice and other small rodents experience during normal ambulation (25, 26). A total of 8 reflective markers were placed on the pendulum system for motion tracking; 4 were fixed to the pendulum arm, and an additional 4 were attached to the base of the system. To determine the coefficient of friction using the passive system, the limb was rotated from its vertical equilibrium position to a starting angle of ∼12 ± 2° (Figure 1B), released, and allowed to oscillate freely, until returning to its equilibrium state. Motion was tracked with a Qualisys AB system at a rate of 60 Hz. Custom MatLab (MathWorks) code was used to calculate the coefficient of friction using a Stanton linear decay model (27, 28). Three trials were recorded for each joint at each time point. These values were then averaged and used for statistical analysis.

The active pendulum was used for cyclic loading. The specimen-mounting system was identical to that in the passive pendulum system. The tube encasing the tibia was placed in the mounting block and fixed with 2 set screws, while the tube containing the femur supported the pendulum arm, which weighed 50 gm (∼2 times the body weight of the mice). The weight was chosen based on our unpublished pilot studies in which efforts were made to approximate forces experienced by the mouse knees during normal walking while accounting for the strength of the bones that would be supporting the joints through 26 cumulative hours of loading. The joint was submerged within a well filled with 35 ml of cell culture medium (29) to hydrate and prolong the viability of the tissue. Testing took place at room temperature, and the medium in the well was replenished as necessary. The pendulum arm was attached to a DC motor via a series of linkages, rotary bearings, and a linear bearing underneath the mounting platform, which drove the active pendulum. The joint was driven through ± 15° of flexion and extension about the resting angle of 70°, at a rate of ∼1.5 Hz (Figure 1B).

### Cyclic loading protocol

**Figure 2:**
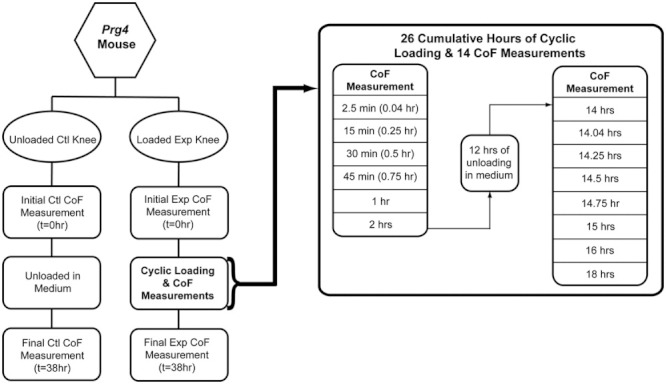
Flow chart of the experimental protocol. Each experimental mouse limb underwent 26 cumulative hours of cyclic loading, 12 hours of unloading in cell culture medium, and oscillation data collection at 16 time points. The control mouse limbs remained in an unloaded state in culture medium for the duration of testing, and oscillation data were collected at 2 time points. Ctl = control; Exp = experimental; CoF = coefficient of friction.

One hind limb from each pair was randomly selected as the experimental knee that would undergo cyclic loading, while the contralateral knee joint was used as the control (Figure 2). Oscillation data were collected with the passive pendulum for each knee (0 minutes) to obtain baseline coefficient of friction values. The control joint was then placed unloaded in culture medium, while the experimental limb was mounted in the active pendulum system for cyclic loading. The experimental joint was cyclically loaded for a period of 2 hours. This time period was chosen based on prior reports that described the effects of cyclic loading on the coefficient of friction of various cartilage specimens using a variety of methods (21, 22, 30). We measured the coefficient of friction frequently throughout the 2-hour loading period, by briefly returning the specimen to the passive pendulum. Following the 2 hours of cyclic loading, the experimental joint was placed unloaded in cell culture medium for 12 hours; this time period was chosen to mirror the murine activity cycle, so that the joint would be able to recover from the initial cyclic loading. After this unloading period, we measured the coefficient of friction and then subjected the joint to an additional 24 hours of cyclic loading to investigate the effect of prolonged cyclic loading. During this second session of cyclic loading, the specimen was again briefly returned to the passive pendulum to measure the coefficient of friction (Figure 2). The coefficient of friction values in the control knees were measured at baseline (0 hours), and then again after 38 hours in culture medium (Figure 2).

### Histologic processing and analysis

Following collection of the final coefficient of friction measurements, experimental and control specimens were placed in 10% buffered formalin for fixation. Specimens were decalcified, embedded in paraffin wax, and cut into 5-μm coronal sections. Sections were then deparaffinized and dehydrated with xylenes and a series of ethanol solutions. Finally, sections were stained with Safranin O, counterstained with fast green, and mounted with a coverslip using Permount (Fisher Scientific). Five observers (EID, GDJ, BCF, LZ, and JJC), who were blinded with regard to *Prg4* genotype and treatment group (cyclically loaded versus unloaded), evaluated the articular cartilage integrity using a scoring system that has been developed and validated specifically for mouse cartilage (3). The scoring system has 3 separate categories: articular cartilage structure (graded on a scale of 0–4), surface layer morphology (graded on a scale of 0–3), and pericellular loss of Safranin O staining (graded on a scale of 0–4), allowing for a maximum total score of 11. Each observer independently scored each tibial plateau, and then the final score was adjusted following a group discussion. Next, scores for each plateau were averaged to obtain 1 score for each category per joint. Category scores were also summed to obtain a total score for each joint. Scores from each category and overall totals were compared with coefficient of friction values to investigate for a direct relationship between articular cartilage surface integrity and the coefficient of friction.

### Chondrocyte viability

Because this study involved prolonged ex vivo testing of murine knee joints, chondrocyte viability was assessed using fluorescein diacetate and propidium iodide (PI) paravital staining. Living and dead cells accumulate fluorescein diacetate and PI, respectively. Prg4^+/+^, Prg4^+/−^, and Prg4^−/−^ specimens (n = 2 pairs per genotype; 2 experimental and 2 control knee joints per genotype) were used to examine chondrocyte viability. Specimens underwent preparation and testing protocols as described above. Following testing, the capsules from both cyclically loaded and control specimens were dissected away, and joints were disarticulated. Using a scalpel, cartilage surfaces were separated from each long bone to ensure that the fluorescein diacetate/PI dye penetrated through the tissue. Next, a scalpel was used to slice a 100–200–μm sagittal section from each femoral condyle. Fluorescein diacetate stock solution was prepared by dissolving 40.1 mg of fluorescein diacetate (Sigma) in 10 ml of acetone, and PI stock solution was prepared by dissolving 100 mg of PI in 10 ml of distilled water. A working solution of fluorescein diacetate was prepared by combining 5 μl of stock solution with 15 ml of phosphate buffered saline (PBS); 10 μl of PI stock solution were combined with 10 ml of PBS and 2.5% EDTA to make a working PI solution. Equal volumes of each working solution were mixed to create the final stain. Cartilage samples were incubated in the final stain in the dark at room temperature for 5 minutes. Following incubation, samples underwent 5 successive washes with PBS. Once the cartilage sections were washed, they were mounted on glass slides with Vectashield mounting medium (Vector), which served to protect the samples against photobleaching. Imaging took place immediately following specimen preparation.

Confocal images were acquired with a Nikon C1si confocal microscope using diode lasers 488 and 561. Serial optical sections were performed with EZ-C1 computer software. Z-series sections were collected at 1.5 μm with a 20× PlanApo lens and a scan zoom of 1×. Each wavelength was acquired separately by invoking frame lambda. Projections were performed in Elements computer software (Nikon). Images were qualitatively assessed for the presence of live chondrocytes.

### Statistical analysis

A normal mixed model on log-adjusted coefficient of friction values was used to assess the effects of time, genotype, and limb (experimental versus control) on the coefficient of friction values. A log-normal mixed model was used to assess the coefficient of friction values across all time points. A significant *P* value of less than 0.05 was set a priori.

A two-way analysis of variance was used to test for statistically significant differences among *Prg4* genotypes and treatment groups within each histologic scoring category and for the overall total score (SigmaStat; Systat Software).

## RESULTS

**Figure 3:**
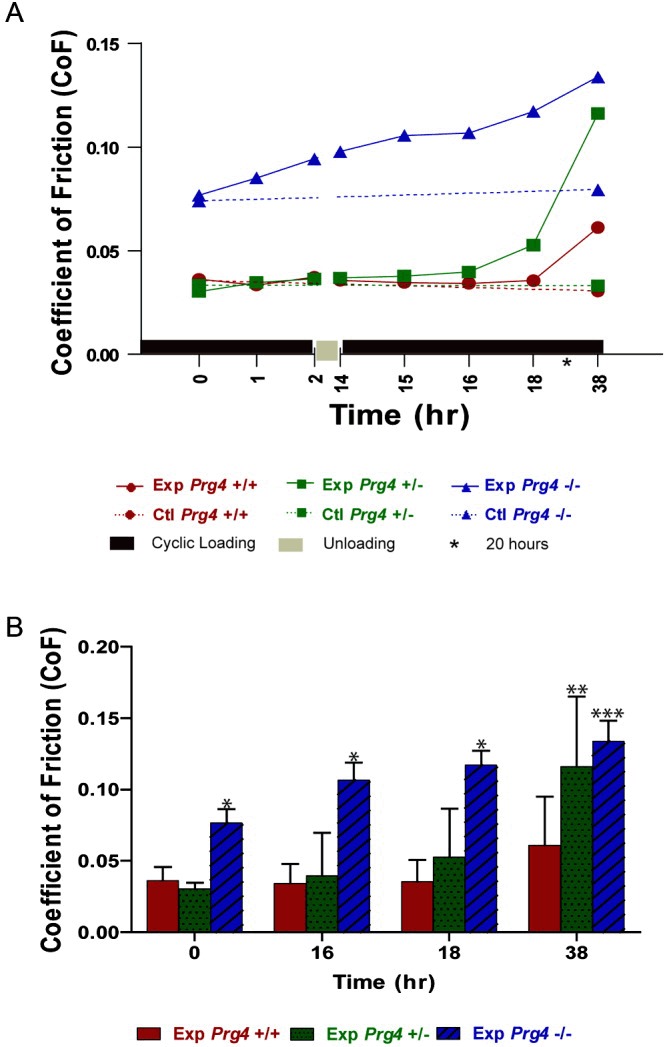
A, Mean coefficient of friction values in experimental (Exp) and control (Ctl) mouse joints over the course of testing. The shaded bars at the bottom represent the time during which the experimental joint was cyclically loaded and unloaded. Note that the control coefficient of friction values did not change during the course of the experiment; however, the control coefficient of friction values in the Prg4^−/−^ mice were significantly higher than those in the other 2 genotypes (*P* < 0.0001). The experimental coefficient of friction values in the Prg4^−/−^ mice rose steadily during the 26 hours of cyclic loading. The coefficient of friction values in the Prg4^+/+^ and Prg4^+/−^ mice were comparable at the onset of cyclic loading; however, at the conclusion of the cyclic loading experiment, the coefficient of friction values in the Prg4^+/−^ mice were closer to those in the Prg4^−/−^ mouse joints than to those in the Prg4^+/+^ mouse joints. B, Coefficient of friction values in cyclically loaded joints from each genotype at 0, 16, 18, and 38 hours. At 0, 16, and 18 hours, the coefficient of friction values in the Prg4^−/−^ mice were significantly higher than those in the wild-type and heterozygous mice. However, at 38 hours, the coefficient of friction values in both the Prg4^−/−^ and Prg4^+/−^ mice were significantly higher than those in the Prg4^+/+^ mice. Bars show the mean ± SD. * = *P* < 0.0001 versus Prg4^+/+^ and Prg4^+/−^ mice; ** = *P* < 0.003; *** = *P* < 0.0001, versus Prg4^+/+^ mice. Color figure can be viewed in the online issue, which is available at http://onlinelibrary.wiley.com/journal/10.1002/(ISSN)1529-0131.

The baseline (0 hours) coefficient of friction values in Prg4^+/+^ mouse joints (mean ± SD 0.036 ± 0.0096) and Prg4^+/−^ mouse joints (0.030 ± 0.0044) did not significantly differ from each other (Figures 3A and B). In contrast, the baseline coefficient of friction in Prg4^−/−^ mouse joints (0.077 ± 0.0095) was significantly higher than that in Prg4^+/+^ and Prg4^+/−^ mouse joints (*P* < 0.001). After 38 hours in culture medium, the coefficient of friction values in unloaded mouse joints did not differ significantly from the coefficient of friction values obtained at baseline for mice with the same *Prg4* genotype (Figure 3A).

Prg4^−/−^ mouse joints exhibited a progressive increase in coefficient of friction values during the first 2 hours of cyclic loading, while no increases in the coefficient of friction were observed in Prg4^+/+^ or Prg4^+/−^ mouse joints during this time frame (Figure 3A). Coefficient of friction changes did not occur when the Prg4^+/+^, Prg4^+/−^, or Prg4^−*/*−^ mouse joints were then unloaded for 12 hours (Figure 3A). However, the coefficient of friction continued to increase in Prg4^−/−^ mouse joints when cyclic loading was resumed, reaching a final mean ± SD coefficient of friction value of 0.13 ± 0.014 (Figures 3A and B). In contrast, there was no change in the coefficient of friction in Prg4^+/+^ mouse joints during the first 6 hours of cyclic loading, but by the end of the experiment, the mean ± SD coefficient of friction had risen to 0.061 ± 0.034. Prg4^+/−^ mouse joints exhibited no change in the coefficient of friction during the first 4 hours of cyclic loading, a mild increase after 6 hours of cyclic loading (0.053 ± 0.034), and an increase to 0.12 ± 0.049 at the end of the experiment (Figures 3A and B). Therefore, although the mean coefficient of friction values in all *Prg4* genotypes rose following 26 total hours of cyclic loading, the increases were significantly greater for Prg4^+/−^ and Prg4^−/−^ mouse joints (*P* = 0.0028 and *P* < 0.0001, respectively) than for Prg4^+/+^ mouse joints (Figure 3B).

**Figure 4:**
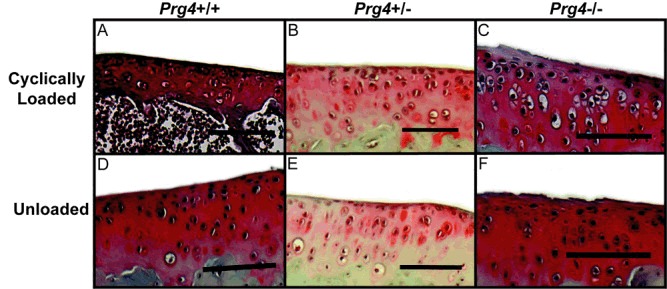
Photomicrographs of mouse knee joints that had the median total histologic score for each *Prg4* genotype and experimental group. A–C, Cyclically loaded Prg4^+/+^, Prg4^+/−^, and Prg4^−/−^ mouse joints with scores of 1.0, 2.5, and 5.0, respectively. D–F, Control (unloaded) Prg4^+/+^, Prg4^+/−^, and Prg4^−/−^ mouse joints with scores of 1.0, 2.0, and 5.0, respectively. Although the coefficient of friction increased significantly in all cyclically loaded joints, this was not accompanied by microscopic evidence of damage that could be attributed solely to loading. Bars = 100 μm; original magnification × 10.

As anticipated (13), Prg4^−/−^ mouse knee joints differed histologically from Prg4^+/+^ mouse knee joints. Unloaded Prg4^−/−^ mouse joints had significantly (*P* < 0.001) higher scores for articular cartilage surface structure and for surface layer morphology compared to unloaded Prg4^+/+^ and Prg4^+/−^ mouse joints, which did not differ from each other. Significant (*P* < 0.001) differences in these measures were also found between the loaded Prg4^−/−^ mouse joints and the loaded Prg4^+/+^ and Prg4^+/−^ mouse joints (Table 1 and Figure 4). Interestingly, within a genotype, joints that had been cyclically loaded for 26 hours did not appear grossly different from their contralateral unloaded controls. However, following loading, Prg4^−/−^ mouse joints, but not Prg4^+/−^ or Prg4^+/+^ mouse joints, had significantly higher surface layer morphology scores compared to their unloaded controls. Lastly, there were no correlations between histologic score and coefficient of friction value.

**Table 1:** Total histologic scores and histologic scores for each category in Prg4 mouse knee cartilage*

	Prg4^+/+^		Prg4^+/−^		Prg4^−/−^	
	Experimental	Control	Experimental	Control	Experimental	Control
Articular cartilage surface structure	0.21 ± 0.33	0.21 ± 0.40	0.40 ± 0.52	0.20 ± 0.26	2.05 ± 0.52†	1.91 ± 0.77†
Surface layer morphology	0.38 ± 0.43	0.30 ± 0.33	0.65 ± 0.47	0.25 ± 0.26	2.09 ± 0.20†‡	1.59 ± 0.86†
Pericellular Safranin O loss	0.88 ± 0.74	0.83 ± 0.65	1.55 ± 0.99	1.40 ± 1.17	1.14 ± 0.60	1.18 ± 0.98
Total	1.46 ± 1.14	1.33 ± 0.96	2.60 ± 1.61	1.85 ± 1.33	5.27 ± 1.06	4.68 ± 2.14

* Values are the mean ± SD. Mouse knee joints were cyclically loaded (experimental) or left unloaded (control).

† *P* < 0.001 versus Prg4^+/+^ and Prg4^+/−^ mouse joint articular cartilage surface structure and surface layer morphology scores (experimental or control condition).

‡ *P* < 0.001 versus control Prg4^−/−^ mouse joint surface layer morphology score.

**Figure 5:**
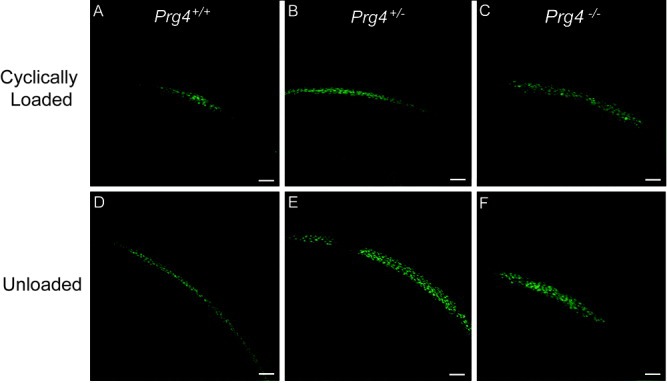
Stacked fluorescence confocal microscopy images of sagittal sections (27.8 ± 12.1 μm) of fluorescein diacetate–stained mouse femoral condyle cartilage samples. Green fluorescence indicates the presence of living cells. A–C, Cyclically loaded Prg4^+/+^, Prg4^+/−^, and Prg4^−/−^ mouse cartilage. D–F, Control (unloaded) Prg4^+/+^, Prg4^+/−^, and Prg4^−/−^ mouse cartilage. Staining and imaging of each sample was performed at the conclusion of the experiment (38 hours). Note that live cells are present in all cartilage specimens and that the density of live cells did not decrease as a consequence of loading. Bars = 50 μm; original magnification × 20.

Although the cyclically loaded and control mouse knee joints were kept in cell culture medium for the entire 38 hours of the experiment, the lack of endogenous blood circulation and the retention of the synovial capsule surrounding the joint limited oxygen and nutrient availability within the joint, which could have led to chondrocyte death. However, all cartilage samples contained living chondrocytes, as assessed by fluorescein diacetate staining (Figure 5).

## DISCUSSION

The objectives of this study were to investigate the effects of cyclic loading on the surface integrity of articular cartilage and on whole-joint frictional properties in mice, and to examine the effects of cyclic loading in wild-type mice (Prg4^+/+^) and mice that are heterozygous (Prg4^+/−^) or homozygous (Prg4^−/−^) for inactivating mutations in *Prg4*, the gene that encodes lubricin.

In contrast to other studies (21, 22, 30), in which significant increases in the coefficient of friction were observed within 2 hours of cyclically loading normal cartilage, in this study, coefficient of friction increases were not observed until at least 6 hours of cyclic loading in wild-type mouse knee joints. This delayed timing of coefficient of friction change may be due to the testing modality, which allowed the articular cartilage to remain undisturbed within an intact synovial capsule. Other studies used articular cartilage that was excised from bovine knees (21, 22, 30). Excision from the intact knee joint could have made these specimens more prone to rapid changes in the coefficient of friction due to weeping of water and glycosaminoglycans from the lateral surfaces or from the loss of water at the cartilage surface due to evaporation. Using medial compartmental bovine knees with and without meniscectomies and a tribologic simulator to apply physiologically relevant (cyclic) loading for 2 hours, McCann et al reported coefficient of friction increases, which were larger in meniscectomized specimens (22). Forster and Fisher investigated the influence of continuous sliding on bovine articular cartilage plugs using a custom-built friction apparatus. They also demonstrated increases in the coefficient of friction over the course of 120 minutes of testing (21). Park et al used bovine cartilage plugs to examine the effect of a sliding motion on the coefficient of friction, as measured by a custom-made friction device and by atomic force microscopy (AFM). Both methods yielded friction increases during the course of the 2-hour experiment (30).

Tribologic testing with cartilage plugs has the advantage of allowing direct isolation and observation of the behavior of articular cartilage and its response to mechanical loading as well as to chemical treatments (23). Hence, when a cartilage plug is cyclically loaded, plowing friction is theoretically eliminated, resulting in a surface friction measurement (24). Coefficient of friction values obtained with AFM are generally higher, often times by an order of magnitude, than values obtained with whole-joint methods or cartilage plugs (3, 30). AFM allows for a characterization of the effects of boundary lubrication on the frictional properties of articular cartilage independent of the interstitial fluid pressurization that is found within diarthrodial joints (3, 24, 30).

Our use of intact mouse knees, while not directly measuring cartilage friction, may more accurately measure the friction within a joint as it functions in vivo. These whole-joint measurements take all of the components of the joint into account, including the menisci, cruciate ligaments, opposing joint surfaces, and joint capsule. Thus, the lubrication mechanisms differ in whole joints, as compared to those found in cartilage plug and AFM specimens, and may contribute to the differences in coefficient of friction response between these specimens (24).

Cyclic loading appears to be the primary cause of the increase in coefficient of friction values in this study, since the unloaded (contralateral control) knees had no change in the coefficient of friction after 38 hours in cell culture medium. In contrast, the coefficient of friction values in cyclically loaded joints increased during the same 38-hour period. The fact that the coefficient of friction increased following cyclic loading is consistent with the findings of previous studies (21, 22). Interestingly, only Prg4^−/−^ mouse joints demonstrated a clear increase in the coefficient of friction following 2 hours of cyclic loading. This increase in the coefficient of friction remained unchanged when the joints were stored unloaded in culture medium for 12 hours, but then the coefficient of friction continued to increase when the cyclic loading resumed.

Lubricin, also referred to as proteoglycan 4 and superficial zone protein, is produced by synoviocytes that line the joint capsule and by chondrocytes within the superficial zone of articular cartilage. Previous work has demonstrated that the absence of lubricin results in protein deposition on cartilage surfaces, the disappearance of superficial zone chondrocytes, and synoviocyte overgrowth (1). Additionally, Prg4^−/−^ mouse knee joints have been shown to develop evidence of cartilage wear by 2 weeks of age (13). The results of the present study confirm the important role of lubricin for protection against load-induced wear. The assays used in this study demonstrated that cyclic loading had no effect on the coefficient of friction in Prg4^+/+^ mouse knee joints loaded for at least 6 hours, whereas a continual increase in the coefficient of friction occurred in Prg4^−/−^ mouse joints over that same period. Intriguingly, the coefficient of friction in Prg4^+/−^ mouse knees at baseline and during the first 4 hours of cyclic loading was indistinguishable from that in Prg4^+/+^ mouse joints. However, by 6 hours of cyclic loading, a difference between Prg4^+/−^ and Prg4^+/+^ mouse knee joints began to emerge. After 26 hours of cyclic loading, Prg4^+/−^ mouse joints had developed coefficient of friction values that were closer to those of Prg4^−/−^ mouse joints rather than Prg4^+/+^ mouse joints. These results suggest that the ability of lubricin to provide long-term protection to joints is dose (i.e., concentration) dependent, which could have important clinical implications.

It is reasonable to question whether an increased coefficient of friction occurring only after 6 hours of cyclic loading in the Prg4^+/−^ joints will be clinically relevant, particularly since there was no difference between Prg4^+/−^ and Prg4^+/+^ mouse joints at baseline or following 4 hours of cyclic loading. It can be speculated that mice and humans who are genetically deficient for one Prg4 allele may synthesize sufficient quantities of lubricin during normal use but be unable to do so during periods of excessive use. Cyclic loading may denude areas of lubricin from the cartilage surface that must be refilled either by newly synthesized lubricin from underlying superficial zone chondrocytes or by the deposition of lubricin from synovial fluid. Continuous passive motion of joints has previously been reported to induce lubricin expression by superficial zone chondrocytes (14), so genetic or acquired diseases that decrease lubricin synthesis and delay lubricin replacement on denuded surfaces could contribute to precocious cartilage wear. Too few heterozygous carriers of CACP mutations have been studied to know whether carriers have increased rates of joint failure compared to noncarriers. However, if insufficient lubricin production is responsible for the rise in the coefficient of friction that occurs following cyclic loading, then therapies aimed at supplementing lubricin or increasing its production may improve long-term outcomes in patients who have a significant reduction in lubricin abundance as a result of injury or illness.

Friction is defined as the resistance of motion between two joint surfaces in contact with one another (24). As the coefficient of friction values presented in this study have demonstrated, cyclic loading alters the frictional properties of articular cartilage. Because the assay used in the present study used excised limbs and occurred over 38 hours, it was important to confirm that the chondrocytes in the tested joints remained alive throughout the study. Viable cells were observed in all specimens by fluorescein diacetate staining; therefore, death is unlikely to account for the coefficient of friction increases that were observed after 26 hours of cyclic loading for all *Prg4* genotypes. These findings were not surprising, since previous studies have shown that cartilage can maintain between 91% and 97% chondrocyte viability after 14 days and between 70% and 83% chondrocyte viability after 28 days when stored in culture medium at 4°C (31, 32). However, one cannot preclude the possibility that this protocol reduced the biosynthetic activity of chondrocytes and synoviocytes in the absence of cell death. It is also worth noting that this study did not analyze the viability of other tissues within the joint, such as the synovium. While viable chondrocytes were found within the specimens, the other tissues within the whole joint could have experienced cell death and contributed to the coefficient of friction increases in the later stages of cyclic loading. Future long-term cyclic studies should investigate the viability of these tissues.

Finally, because the friction measurements were performed on intact joints, there is a possibility that tissues other than the articular cartilage contributed to the increase in the coefficient of friction. Twenty-six hours of cyclic loading could have altered the joint capsule as well as the intracapsular ligaments. Additionally, the use of mice in this biomechanical study posed design challenges due to the small size of the specimens. However, achieving the construction of a reliable and reproducible ex vivo cyclic loading apparatus for mouse knee joints could be useful since many mouse strains with mutations affecting cartilage development and homeostasis have been generated.
